# Near infrared fluorescence imaging of EGFR expression *in vivo* using IRDye800CW-nimotuzumab

**DOI:** 10.18632/oncotarget.23557

**Published:** 2017-12-21

**Authors:** Wendy Bernhard, Ayman El-Sayed, Kris Barreto, Carolina Gonzalez, Wayne Hill, Angel Casaco Parada, Humphrey Fonge, C. Ronald Geyer

**Affiliations:** ^1^ Department of Pathology, University of Saskatchewan, Saskatoon, Canada; ^2^ Center for Molecular Immunology, Havana, Cuba; ^3^ Department of Medical Imaging, University of Saskatchewan, Saskatoon, Canada; ^4^ Department of Medical Imaging, Royal University Hospital, Saskatoon, Canada; ^5^ Saskatchewan Centre for Cyclotron Sciences (SCCS), Fedoruk Centre, Saskatoon, Canada

**Keywords:** nimotuzumab, near infrared fluorescence imaging, image-guided surgery, epidermal growth factor receptor, IRDye800CW

## Abstract

Nimotuzumab is a humanized anti-epidermal growth factor receptor (EGFR) monoclonal antibody that is approved in many countries for the treatment of EGFR-positive cancers. Near infrared (NIR) fluorescent dye-labeled antibodies represent an attractive class of image-guided surgical probes because of their high specificity, tumor uptake, and low dissociation from tumor cells that express the antigen. In this study, we developed a NIR fluorescent dye-labeled nimotuzumab immunoconjugate, IRDye800CW-nimotuzumab, and evaluated *in vitro* binding with EGFR-positive cells, *in vivo* tumor uptake by NIR fluorescent imaging, and *ex vivo* biodistribution. There was no difference in binding between nimotuzumab and IRDye800CW-nimotuzumab to EGFR-positive cells. In mice bearing EGFR-positive xenografts, IRDye800CW-nimotuzumab uptake peaked at 4 days post injection and slowly decreased thereafter with high levels of accumulation still observed at 28 days post injection. In EGFR-positive xenografts, IRDye800CW-nimotuzumab showed more than 2-fold higher uptake in tumors compared to IRDye800CW-cetuximab. In addition, liver uptake of IRDye800CW-nimotuzumab was two-fold lower than cetuximab. The lower liver uptake of IRDye800CW-nimotuzumab could have implications on the selected dose for clinical trials of the immunoconjugate. In summary, this study shows that nimotuzumab is a good candidate for NIR fluorescent imaging and image-guided surgery.

## INTRODUCTION

Epidermal growth factor receptor (EGFR) is a 170 kDa cell surface glycoprotein belonging to the type-1 tyrosine kinase receptor subfamily [1]. EGFR is involved in cell proliferation, differentiation, and survival and is often overexpressed, amplified, or mutated in a number of different cancers [2]. Overexpression of EGFR has been implicated in all aggressive cancers of epithelial origin, including squamous cell head and neck (90–100%) [3], glioma (90–100%) [4], non-small cell lung (75–90%), colorectal (80–85%) [5], breast (20–30%) [6], and cervical (87–100%) [7].

Currently, resection of tumor tissue relies on images taken before surgery, either by computed tomography (CT), positron emission tomography (PET), or magnetic resonance imaging (MRI), followed by unassisted surgery using white light illumination and visual inspection. This leads to a high risk of tumor recurrence due to ill-defined tumor margins and inadequate resection [8]. Use of new technologies such as neuronavigation and intraoperative CT or MRI have increased in recent years, however, evidence of the advantage of these technologies over conventional techniques remains scarce. In addition, these tools are not antigen specific, do not significantly improve tumor visualization, and are not performed in real time. Intraoperative fluorescence probes offer improved sensitivity and tumor specificity and can be used during surgery thereby improving surgical outcomes [9].

Image-guided surgery is a recently developed technique that is used to specifically target tumors with fluorescent labels to more precisely identify cancer cells, aiding surgeons in defining tumor margins and completely removing tumors during resection [10]. In a multicenter randomized phase III trial, Stummer *et al.*, [11] compared two types of surgical resection for glioma followed by standard radiotherapy. Patients with malignant tumors were randomly allocated to receive oral 5-aminolevulinic acid (*in situ* fluorescent dye) before surgery and resection of fluorescent tissue or to conventional microsurgery with white light. Fluorescence-guided surgery led to a higher number of completely resected tumors than white-light guided surgery (65% *vs* 36%) and the 6-month progression-free survival was two-fold higher using fluorescence (41%) compared to white light (21%). These results show the importance of delineating tumor margins during resection, however, 5-aminolevulinic acid relies on the high metabolism of cancer cells to produce the fluorescence. Targeted approaches using fluorescent molecules that specifically bind cancer cells are needed to determine tumor margins and provide quantitative information about EGFR expression.

Monoclonal antibodies can target cancer specific antigens on the cell surface and can be conjugated to CT contrast agents, paramagnetic particles, microbubbles, multimodality probes, radioisotopes, fluorophores, and other probes to image tumors [12]. A number of fluorophores, which are well tolerated in humans, are used in image-guided surgery, [12], including Cy5/7 dyes, methylene blue (MB), 5-aminolevulinic acid (5-ALA), indocyanine green (ICG), fluorescein (FITC), IRDye700, and IRDye800 [12]. MB, 5-ALA, and ICG function as passive probes that have non-specific uptake in tumors [12]. FITC and IRDye700/800 can be conjugated to antibodies. IRDye800CW is available in good manufacturing practice (GMP) formulations for human use [12].

There are two major obstacles in using fluorophores for imaging: auto-fluorescence from human tissue and limited tissue penetration [13]. These issues can be overcome by using NIR fluorescent dyes that have emission wavelengths with reduced auto-fluorescence [13] and have tissue penetration of < 1 cm for fluorescence reflectance imaging (FRI) and < 10 cm for fluorescence molecular tomography (FMT), [13]. NIR dyes are also useful in real-time endoscopy or during surgery where the wound bed is exposed.

IRDye800 is an NIR fluorophore [12] that has been conjugated to antibodies to target tumors, including IRDye800CW-cetuximab [14] and IRDye800CW-panitumumab [15] for head and neck cancer and IRDye800CW-trastuzumab for breast cancer [16]. IRDye800CW-cetuximab is currently in clinical trials for imaging and image-guided surgery for head and neck cancer, malignant glioma, and pancreatic cancer. IRDye800CW-Bevacizumab is clinical trials for imaging and image-guided surgery of breast cancer and colorectal cancer [17].

Nimotuzumab is an anti-EGFR antibody with binding characteristics that make it desirable as a molecular-targeted imaging probe. First, it has minimal transient binding to low EGFR-expressing healthy tissues such as the skin [18]. Garrido *et al.*, [18] showed that nimotuzumab requires bivalent binding for stable attachment to the cell surface and that the low skin toxicity of nimotuzumab is attributed to its transient monovalent binding in low-EGFR expressing tissues. This low transient monovalent binding is due to a 10-fold lower affinity of nimotuzumab for EGFR compared to cetuximab or panitumumab [18]. Second, nimotuzumab has limited toxicity compared to EGFR antibodies cetuximab and panitumumab, which have significant cutaneious toxicity [19]. Third, nimotuzumab is approved for head and neck cancer, glioma, and nasopharageal cancer in several countries [20]. There are also more than 60 clinical trials ongoing or completed using nimotuzumab for cancer therapy [17].

In this study, we evaluated IRDye-800CW-nimotuzumab as a NIR fluorescent imaging probe and compared it to IRDye-800CW-cetuximab. We showed that IRDye-800CW-nimotuzumab binds to a variety of EGFR positive human cancer cell lines *in vitro* and *in vivo*. Biodistribution studies showed very little non-specific IRDye-800CW-nimotuzumab organ uptake and IRDye-800CW-nimotuzumab accumulated primarily in the tumor after 48 hours post injection (hpi). Tumor uptake of IRDye-800CW-nimotuzumab was comparable to IRDye800CW-cetuximab, which is currently in clinical trials for image-guided surgery for head and neck cancer, pancreatic cancer, and glioma.

## RESULTS

### Conjugation and characterization of IRDye800CW-labeled antibody conjugates

IRDye800CW was conjugated to nimotuzumab (anti-EGFR) and a control antibody (anti-maltose binding protein) non-specifically through free primary amines with NHS chemistry. Conjugated and unconjugated antibodies were analyzed for purity, size, and binding to recombinant EGFR. Nimotuzumab, the control antibody, and the IRDye800CW-immunoconjugates were ≥ 95% pure and had the expected molecular weight (Supplementary Figure 1). IRDye800CW-nimotuzumab but not the control antibody bound to recombinant human EGFR with a K_D_ of 20.1 ± 0.8 nM, which was not significantly different than the K_D_ of unlabeled nimotuzumab, 22.3 ± 2.3 nM (*p* value > 0.05) (Supplementary Figure 2).

### *In vitro* characterization and serum stability of nimotuzumab and IRDye800CW-nimotuzumab

**Figure 1:**
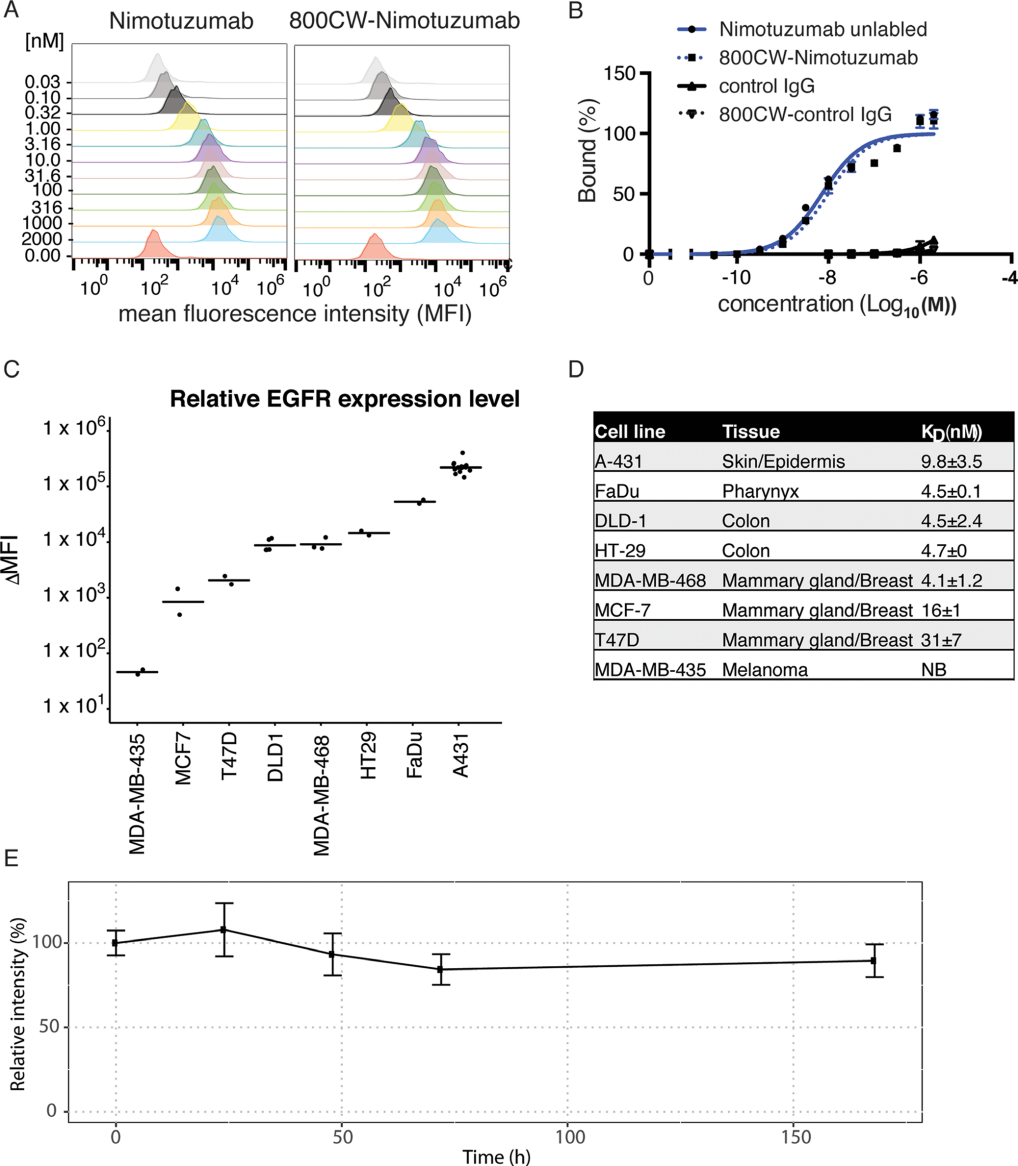
Nimotuzumab binding to cell lines (**A**) Nimotuzumab and IRDye800CW-nimotuzumab were titrated against DLD-1 cells and analyzed by flow cytometry using a FITC-labeled secondary antibody. (**B**) Titration curves of nimotuzumab, IRDye800CW-nimotuzumab, and control IgG against DLD-1 cells showing percent bound against antibody concentration. (**C**) Nimotuzumab binding to cell lines expressing various levels of EGFR. The change in mean fluorescence intensity (ΔMFI) is plotted for each cell line. (**D**) Summary of cell lines tested for nimotuzumab binding and their relative K_D_ value. (**E**) IRDye800CW-nimotuzumab stability was assayed in mouse serum at 37°C over the course of seven days. NB represents no binding. Error bars represent standard deviation.

We characterized the binding of nimotuzumab and IRDye800CW-labeled nimotuzumab to the EGFR-positive cell lines DLD-1 or A431 cells using flow cytometry (Figure 1A, 1B, Supplementary Figure 3). A slight increase in the K_D_ was observed for IRDye800CW-nimotuzumab relative to unlabeled nimotuzumab (*p* value > 0.05). IRDye800CW-nimotuzumab had a K_D_ of 9.7 ± 1.6 nM and 10.4 ± 1.3 nM on DLD-1 and A431 cells, respectively, whereas nimotuzumab had a K_D_ of 4.5 ± 2.4 nM and 9.8 ± 3.5 nM on DLD-1 and A431 cells, respectively. The control antibody had negligible cell binding ( < 5%) (Figure 1B).

Next, we characterized the affinity and the relative amount of EGFR per cell on various cancer cell lines. The level of nimotuzumab binding in these cell lines was used to approximate EGFR expression. Using flow cytometry, we measured the change in mean fluorescence intensity (ΔMFI) of nimotuzumab to classify cell lines into four groups based on EGFR expression: High - A-431 and FaDu; medium - HT-29, DLD-1 and MDA-MB-468; low - MCF7 and T-47D; and EGFR-negative - MDA-MB-435. There was no statistical significance in EGFR expression between different cell lines within each group but there was significant statistical difference between each group (p-value < 0.0001) (Figure 1C).

The K_D_ of nimotuzumab binding to each cell line was determined using flow cytometry (Figure 1D). Despite having the highest EGFR expression, A-431 cells had a higher K_D_ than the medium EGFR expressing cell lines (Figure 1D). Two cell lines MCF7 and T47D with lower EGFR expression showed significantly weaker binding. The K_D_ for nimotuzumab binding to A-431 was significantly higher (*p* < 0.05) when compared to FaDu, MDA-MB-468, DLD-1, and HT-29. K_D_ values of nimotuzumab binding to MCF7 and T-47D were statistically significantly higher (*p* < 0.01) than FaDu, MDA-MB-468, DLD-1 and HT-29. These results showed that affinities of nimotuzumab to cell lines were correlated with receptor density, except when EGFR expression was extremely high as was seen in A-431 cells.

The serum stability of IRDye800CW-nimotuzumab was measured by incubating IRDye800CW-nimotuzumab in mouse serum for one week. 89±10% of IRDye800CW-nimotuzumab remained intact at 7-days (Figure 1E, Supplementary Figure 4).

### *In vivo* near infrared imaging

**Figure 2:**
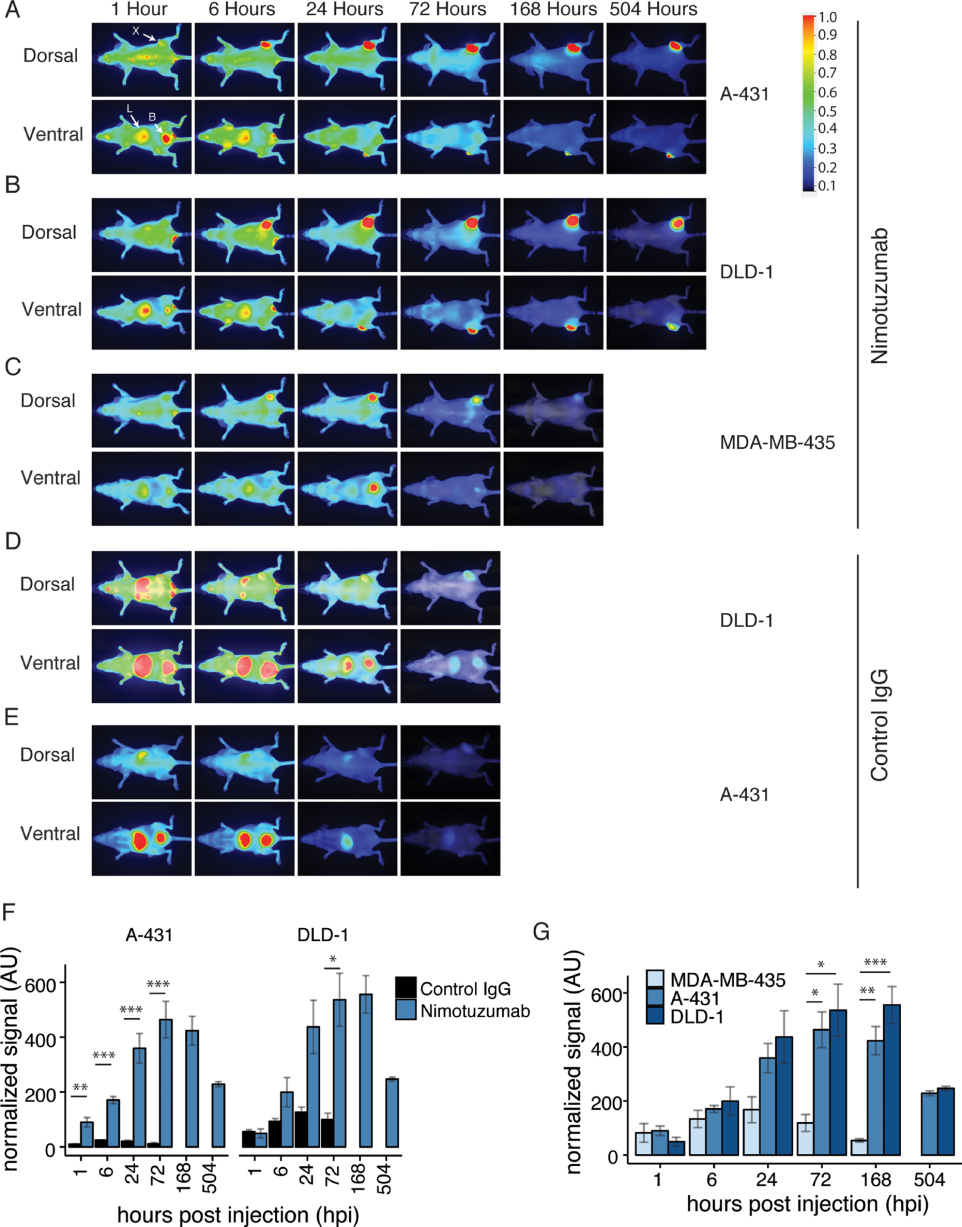
Near infrared imaging of IRDye-800CW-nimotuzumab (**A**–**C**) Mice were intravenously injected with IRDye-800CW-nimotuzumab and fluorescent images were taken of mice bearing (A) A-431, (B) DLD-1, (C) MDA-MB-435 xenografts. Images shown were taken at 1, 6, 24, 72, 168 (7 days), and 504 hours post injection (hpi) (21 days), except MDA-MB-435, which was not imaged at 21 days after injection. (**D**, **E**) IRDye800CW-control IgG was injected into mice bearing DLD-1 and A-431xenografts. Fluorescent images shown were taken after 1, 6, 24, and 72 hpi. The fluorescent scale is shown on the right. All images were normalized to the antibody labeling ratio. X indicates xenograft, L indicates liver, and B indicates bladder. (**F**) Normalized fluorescent signal comparing IRDye800CW-nimotuzumab and the control IgG in A-431 and DLD-1 xenografts at 1, 6, 24, 168, and 504 hpi. (**G**) Normalized fluorescent signal comparing IRDye800CW-nimotuzumab in MDA-MB-435, A-431, and DLD-1 xenografts. ^*^ = *p* value < 0.05. ^**^ = *p* value < 0.01, ^***^ = *p* value < 0.001.AU represents arbitrary units. Error bars represent standard error of the mean.

IRDye800CW-nimotuzumab and IRDye800CW-control antibody were used to image mice bearing A-431, DLD-1, and MDA-MB-435 xenografts, which are high, medium, and low EGFR expressing cell lines, respectively (Figure 2A–2E). A-431 is an epidermoid carcinoma with the highest EGFR expression where DLD-1 is a Duke’s type C. colorectal adenocarinoma with intermediate EGFR expression. MDA-MB-435 cells were used as a negative control as they had no detectable EGFR expression (Figure 2C). The control antibody was tested in A-431 and DLD-1 cell lines (Figure 2D, 2E).

Mice were imaged in dorsal and ventral positions. Dorsal images showed minimal kidney uptake of IRDye800CW-nimotuzumab and long residence time in the xenograft in both A-431 and DLD-1 xenografts with tumor accumulation observed up to 21 days (Figure 2A, 2B). Ventral images showed some clearance through the bladder and fast liver clearance of IRDye800CW-nimotuzumab in the first few hpi, with a significant decrease after 24 hpi. In A431 xenografts the fluorescent signal of IRDye800CW-nimotuzumab was significantly higher than the IRDye800CW-control IgG at all time points (*p* < 0.01 for 1 hour, *p* < 0.001 for other time points) (Figure 2F). In DLD-1 xenografts there was no significant difference in the fluorescent signal between IRDye800CW-nimotuzumab and the IRDye800CW-control IgG up to 24 hpi (*p* > 0.05) (Figure 2F). By 72 hpi IRDye800CW-nimotuzumab was significantly higher in DLD-1 xenografts compared to the IRDye800CW-control antibody (*p* < 0.05).

When different xenografts treated with IRDye800CW-nimotuzumab were compared, accumulation of IRDye800CW-nimotuzumab in A-431 (high EGFR expression) and DLD-1 (intermediate EGFR expression) cells was not significantly different at any of the imaging time points (*p* > 0.05) (Figure 2G). However, IRDye800CW-nimotuzumab accumulation in A-431 and DLD-1 cells was significantly higher than accumulation in EGFR negative (MDA-MB-435) xenografts after 72 hpi (*p* < 0.05 at 72 hpi for both A-431 and DLD-1, *p* < 0.01 at 168 hpi for DLD-1 and *p* < 0.001 at 168 for A-431) (Figure 2F). These results showed that IRDye800CW-nimotuzumab was specific for EGFR expression, but EGFR expression alone did not correlate with increased signal.

The serum stability of IRDye800CW-nimotuzumab indicated that it would be stable *in vivo*. To confirm that the fluorescence observed in the xenograft image was not due to free IRDye800CW, we imaged A-431 xenografted mice injected with quenched IRDye800CW (Supplementary Figure 5). IRDye800CW distributed quickly throughout the mice within 1 hpi and by 6 hpi the dye cleared through the bladder in ventral images. At 24 hpi, only a very faint signal was observed in the xenograft.

During imaging experiments, we did not observe any adverse effects on the mice from administering IRDye800CW-nimotuzumab. In these experiments, no mice died or showed signs of significant weight loss (Supplementary Figure 6), or other gross abnormalities.

### *Ex vivo* imaging and biodistribution

**Figure 3:**
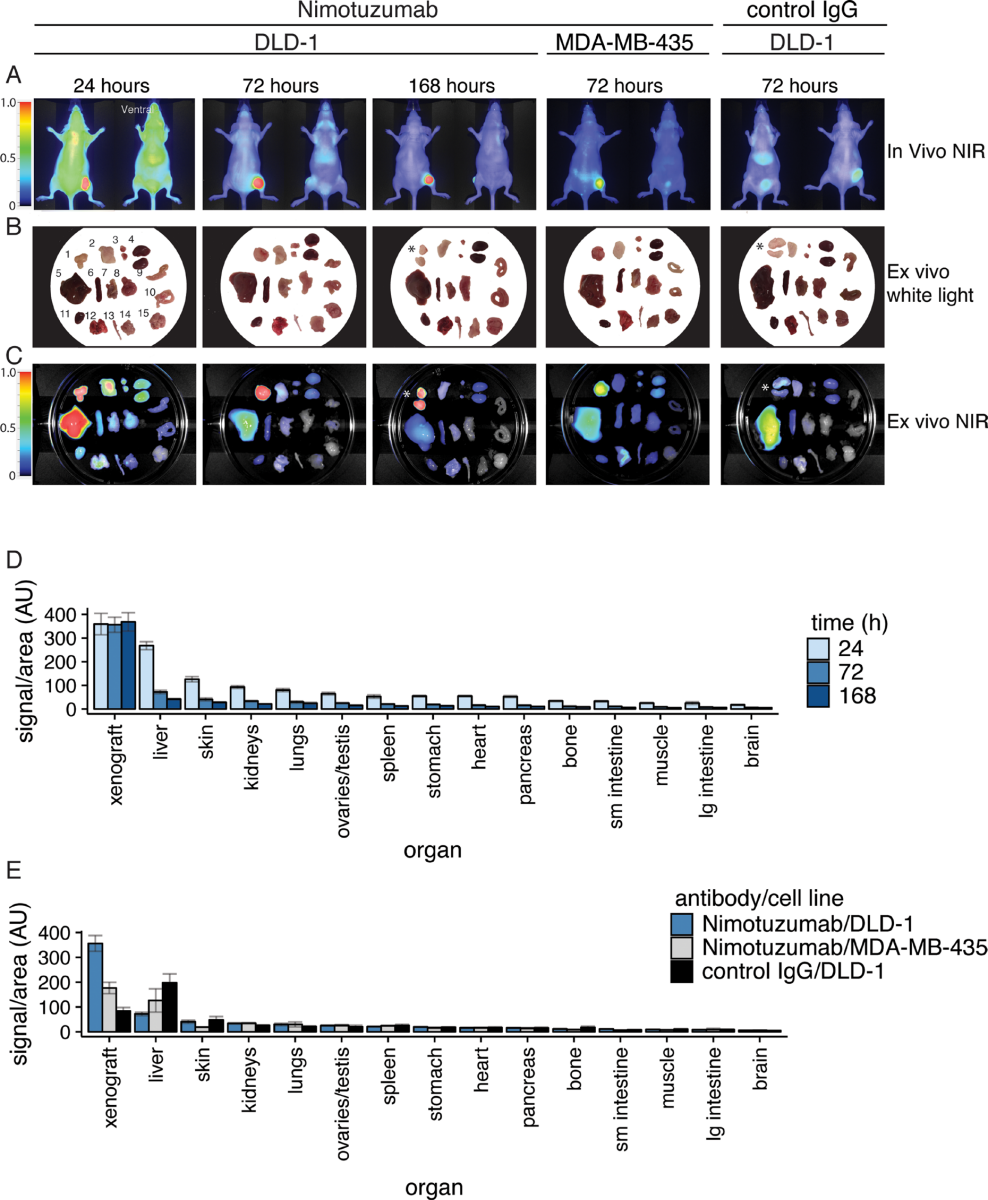
Biodistribution analysis of IRDye800CW-nimotuzumab in mice bearing DLD-1 and MDA-MB-435 xenografts (**A**) Mice bearing DLD-1 and MDA-MB-435 xenografts were intravenously injected with IRDye800CW-nimotuzumab. Fluorescent images shown were taken at 24, 72, and 168 hours post-injection (hpi) for DLD-1 and 72 hpi for MDA-MB-435. Control IgG was injected into mice bearing DLD-1 xenografts. Images were taken at 72 hpi. Organs were collected at these time points. (**B**) White light images and (**C**) fluorescent images were taken as shown. The fluorescent signal was calculated from organs collected from mice, and based on two areas measured from images of each organ in C. 1 = tumor, 2 = skin, 3 = ovaries, 4 = kidneys, 5 = liver, 6 = spleen, 7 = pancreas, 8 = stomach, 9 = large intestine, 10 = small intestine, 11 = heart, 12 = lungs, 13 = bone, 14 = muscle, 15 = brain. (**D**) Fluorescent signal of organs from mice bearing DLD-1 xenografts injected with IRDye800CW-nimotuzumab collected at 24, 72, and 168 hpi. ^*^ respresents xenografts that have been sliced into segments. (**E**) Fluorescent signal of organs from mice injected with IRDye800CW-nimotuzumab bearing DLD-1 or MDA-MB-435 xenograft, or control IgG bearing DLD-1 xenografts collected at 72 hpi. AU is arbitrary units. Error bars represent standard error of the mean.

We performed biodistribution analysis of IRDye800CW-nimotuzumab at 24, 72, and 168 hpi in mice bearing DLD-1 xenografts (Figure 3). Biodistribution was also quantified at 72 hpi in EGFR-negative xenograft MDA-MB-435 and for the IRDye800CW-control antibody in DLD-1 xenografts. *In vivo* NIR images were taken of mice (Figure 3A) and *ex vivo* white light and NIR images of dissected organs (Figure 3B, 3C). Consistent with the *in vivo* imaging, fluorescence is mostly visible in the liver, tumor, and kidney at 24 hpi. Additionally, there was low uptake in the skin, ovaries/testes, lungs, heart, spleen, pancreas, and stomach. After 72 hpi, the fluorescence was cleared from most organs except for the tumor and to a lesser extent the liver, skin, and kidneys. At 168 hpi fluorescence was only visible in the tumor and to a lesser extent in the liver.

NIR *ex vivo* images of organs collected from five mice bearing DLD-1 xenografts injected with IRDye800CW-nimotuzumab were used to calculate the signal per area for each organ at 24, 72, and 168 hpi (Figure 3D). At all time points, the signal was highest in the tumor with average fluorescent values of 358 ± 45, 356 ± 32, 368 ± 39 AU, at 24, 72, and 168 hpi, respectively. Low uptake was observed in the liver, skin, kidneys, and lungs and these decreased to background values at 168 hpi. At 72 hpi the highest uptake was observed for IRDye800CW-nimotuzumab in EGFR-positive DLD-1 xenograft (356 ± 32 AU) compared to EGFR-negative MDA-MB-435 (177 ± 23 AU) and IRDye800CW-control antibody in DLD-1 (84 ± 14 AU) (Figure 3E).

**Figure 4:**
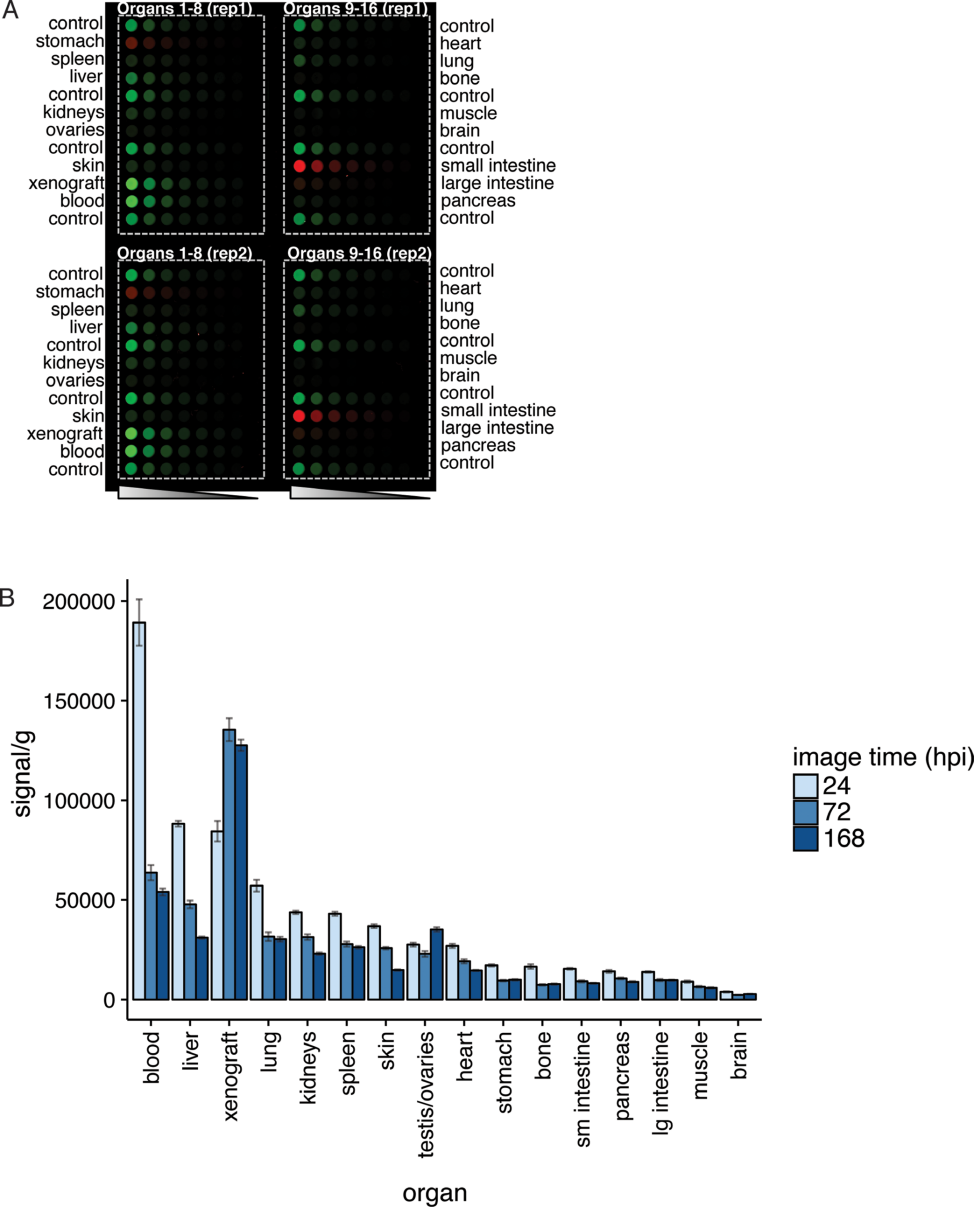
Homogenization of tissues for biodistribution analysis (**A**) Tissues and organs from mice injected with IRDye800CW-nimotuzumab or control IgG bearing DLD-1 xenografts were homogenized, diluted, and the fluorescence measured. (**B**) The fluorescent signal measured per gram of organ was plotted at 24, 72, and 168 hpi. Error bars represent standard error of the mean.

Organs collected for biodistribution were homogenized to analyze the amount of IRDye800CW-nimotuzumab per organ weight (Figure 4A). The blood, xenograft, and liver had the highest signal in the 800 nm channel, which was consistent with the *ex vivo* analysis (Figure 3D, 3E) for the xenograft and liver. Signal per gram was calculated (Figure 4B) based on the fluorescence signal measured from the homogenized organs and tissues. Blood had the highest signal at 24 hpi followed by the liver and xenograft. After 72 hpi the fluorescence signal in the blood and liver decreased whereas the xenograft increased. After 168 hpi the blood and liver further decreased to near background and the xenograft remained high. All other organs and tissues tested had fluorescence signal at background levels (Figure 4B). This analysis gave similar results to what was observed in the *ex vivo* analysis (Figure 3D) and were consistent with the *in vivo* imaging showing IRDye800CW-nimotuzumab accumulating in the tumor with low background accumulation in other organs.

### Comparison of IRDye800CW-nimotuzumab to IRDye800CW-cetuximab

**Figure 5:**
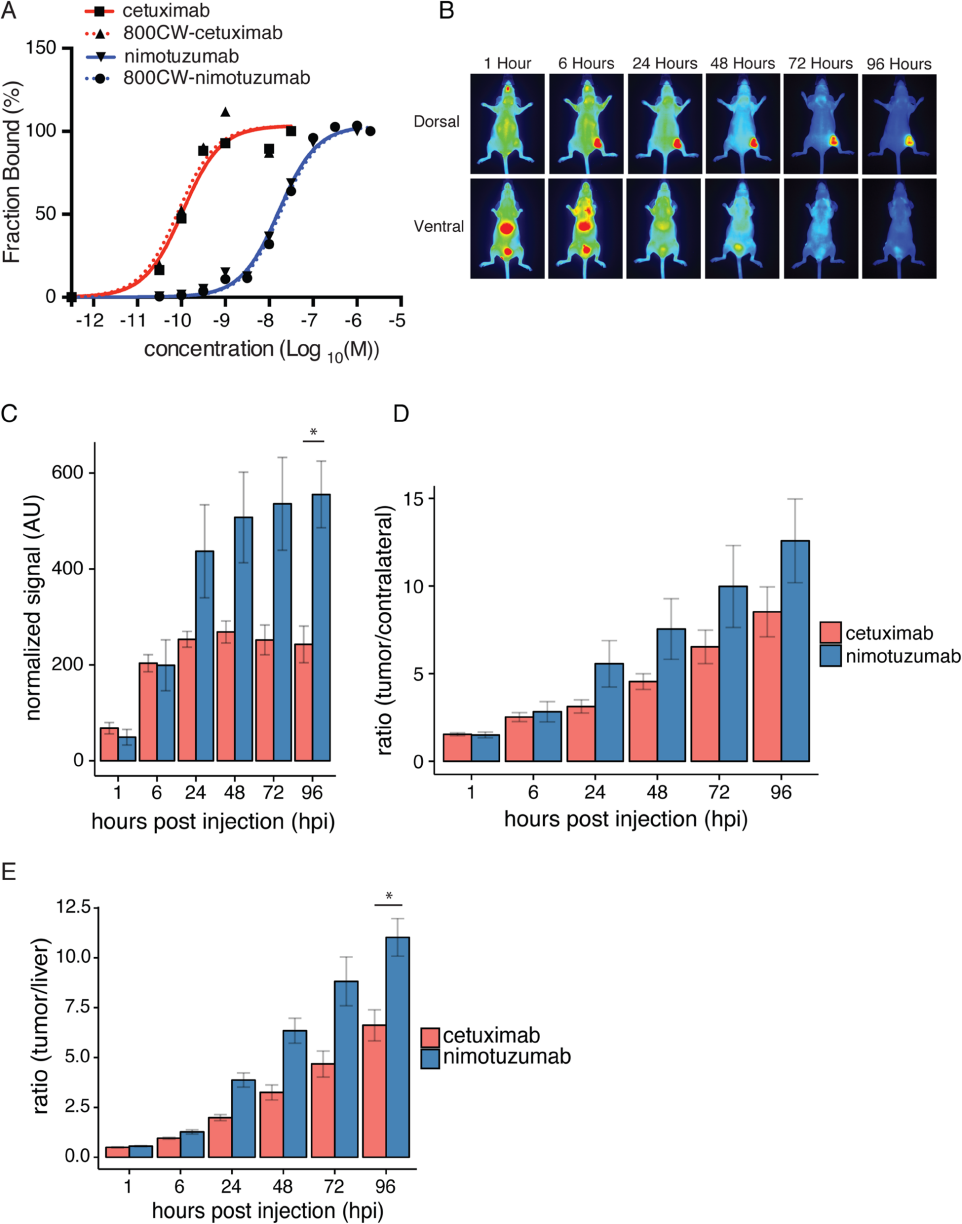
Comparison of nimotuzumab-IRDye800CW and cetuximab-IRDye800CW (**A**) Nimotuzumab, IRDye800CW-nimotuzumab, cetuximab, and IRDye800CW-cetuximab titrated against A-431 cells. (**B**) Fluorescent images of mice bearing DLD-1 xenografts, injected with cetuximab-IRDye-800CW after 1, 6, 24, 48, 72, 96 hpi. (**C**) Normalized fluorescent signal for cetuximab and nimotuzumab over time in DLD-1 xenografts. (**D**) Ratio of tumor to contralateral fluorescent signal for nimotuzumab and cetuximab in DLD-1 xenografts. (**E**) Tumor to liver ratio of fluorescent signal for nimotuzumab and cetuximab in DLD-1 xenografts. ^*^ = *p* value < 0.05. Error bars represent standard error of the mean.

We next compared EGFR-binding and xenograft imaging of IRDye800CW-nimotuzumab to IRDye800CW-cetuximab, an anti-EGFR fluorescent imaging probe currently in clinical trials. Cetuximab was conjugated to IRDye800CW and binding was measured using flow cytometry and compared to IRDye800CW-nimotuzumab and unlabeled antibodies (Figure 5A). There was no significant difference in binding between the IRDye800CW-cetuximab and unlabeled cetuximab (82 ± 8 pM vs 100 ± 34 pM, *p* > 0.5). Cetuximab had a significantly lower K_D_ ∼ 80 pM compared with nimotuzumab (4-10 nM, *p* < 0.0001). These results are similar to previously published K_D_ values for cetuximab of 100 pM [21].

IRDye800CW-cetuximab was imaged in mice bearing DLD-1 xenografts (Figure 5B) and compared to IRDye800CW-nimotuzumab (Figure 5C–E). The uptake of IRDye800CW-cetuximab and IRDye800CW-nimotuzumab in DLD-1 xenografts at 6 hpi was similar, 203 ± 18 AU for cetuximab and 199 ± 53 AU for nimotuzumab, respectively (Figure 5C). IRDye800CW-cetuximab peaked at 48 hpi (fluorescent signal = 269 ± 16 AU) (Figure 5C), whereas IRDye800CW-nimotuzumab peaked at 96 hpi with a 2-fold higher fluorescent signal (555 ± 70 AU) (Figure 5C). IRDye800CW-nimotuzumab was not significantly higher than IRDye800CW-cetuximab until 96 hpi where the fluorescent signal of IRDye800CW-nimotuzumab was significantly higher than IRDye800CW-cetuximab (*p* < 0.05). Ratios of fluorescence of the tumor to contralateral were calculated for IRDye800CW-nimotuzumab and IRDye800CW-cetuximab (Figure 5D). Tumor to contralateral ratios for both IRDye800CW-nimotuzumab and IRDye800CW-cetuximab were above 2 at 6 hpi (Figure 5D) and increased up to 96 hpi, where the ratio for IRDye800CW-nimotuzumab was 12.6 ± 2.4 and IRDye800CW-cetuximab was 8.5 ± 1.4. Tumor to liver ratios for IRDye800CW-nimotuzumab and IRDye800CW-cetuximab were above 2 after 24 hpi (Figure 5E). Tumor to liver ratios for both IRDye800CW-nimotuzumab and IRDye800CW-cetuximab increased up to 96 hpi. IRDye800CW-nimotuzumab had a significantly higher tumor to liver ratio at 96 hpi at 11 ± 0.9 compared to 6.6 ± 0.8 for IRDye800CW-cetuximab (*p* < 0.05) (Figure 5E).

**Figure 6:**
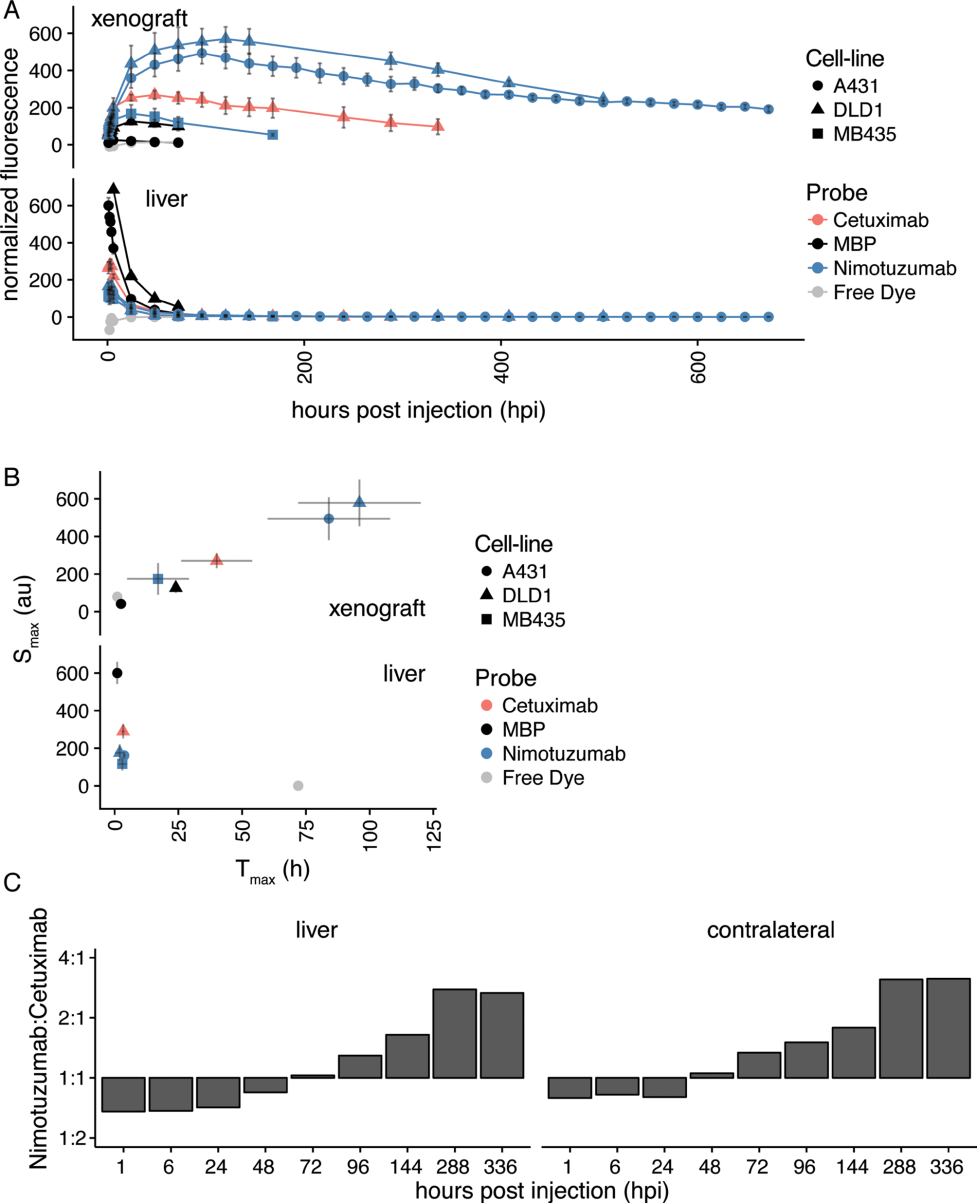
Summary of IRDye800CW-nimotuzumab fluorescent imaging (**A**) The average fluorescent signal in tumor and liver from mice bearing DLD-1, A-431, and MDA-MB-435 xenografts injected with IRDye800CW-nimotuzumab, IRDye800CW cetuximab, and IRDye800CW-control IgG at different times post injection. (**B**) The maximum fluorescent signal (S_max_) in mice bearing DLD-1, A-431, and MDA-MB-435 xenografts at the maximum time (T_max_) post injection of IRDye800CW-nimotuzumab, IRDye800CW-cetuximab and IRDye800CW-control IgG. (**C**) The ratio of fluorescent signal of IRDye800CW-nimotuzumab to IRDye800CW-cetuximab in liver and contralateral over time. Error bars represent standard error of the mean.

A comparison of all antibodies and cell-lines used in this study is shown in Figure 6. In the xenograft, controls (IRDye800CW-MBP IgG and IRDye800CW-nimotuzumab in MDA-MB-435) were found clustered together, IRDye800CW-nimotuzumab in the A-431 and DLD-1 cells were found clustered together (Figure 6A). IRDye800CW-cetuximab was between controls and IRDye800CW-nimotuzumab. In the liver, there was some accumulation of the control IgG and of IRDye800CW-cetuximab to a much lesser extent. The fluorescent signal of IRDye800CW-nimotuzumab peaks later (T_max_ = 96 ± 14 hpi) than IRDye800CW-cetuximab (T_max_ = 40 ± 8 hpi) and has a higher maximum accumulation (S_max_) in the xenograft (Figure 6A, 6B). IRDye800CW-cetuximab has previously been shown to peak at 48 hpi [14]. The difference between IRDye800CW-nimotuzumab and IRDye800CW-cetuximab in the maximum peak and time could be explained by their differences in clearance. IRDye800CW-cetuximab was found to clear through the liver more quickly than IRDye800CW-nimotuzumab, at early time-points up to 1.5-fold more IRDye800CW-cetuximab was in the liver than IRDye800CW-nimotuzumab (Figure 6C). This also reduced the circulating time of IRDye800CW-cetuximab, where there was more IRDye800CW-nimotuzumab in circulation (contralateral) then cetuximab after 48 hpi (Figure 6C). This delayed clearance resulted in significant signal in the tumor for up to 28 days, which allows the tumor to be imaged over an extended period of time if necessary. This also gives surgeons more time to perform resection of the tumor.

## DISCUSSION

Nimotuzumab is approved in a number of countries for squamous cell carcinoma of the head and neck, glioma, and nasopharyngeal cancer [20]. There are 65 clinical trials with nimotuzumab on clinicaltrials.gov that cover a number of cancers, including esophageal, head and neck, lung, cervical, brain, colorectal, stomach, and pancreas. There is a need for fluorescent imaging probes for these and other EGFR positive cancers that can be used for image-guided surgery. In this study, we showed that IRDye800CW-nimotuzumab binds to EGFR positive cell lines *in vitro* and *in vivo* and showed fluorescent imaging properties that make it an excellent candidate for repurposing as an imaging probe. A benefit of using therapeutic molecules for imaging is that many of the side effects and toxicities are already established. Cetuximab, for example has been repurposed for fluorescent imaging and is in phase I and phase II clinical trials for malignant glioma and pancreatic cancer imaging and fluorescence-guided surgery with IRDye-800CW [17]. Nimotuzumab is safe and well tolerated in the clinical setting and should show less side effects for imaging as the imaging dose is significantly lower than the therapeutic dose.

There are three FDA approved EGFR monoclonal antibodies, cetuximab, panitumumab, and necitumumab. Necitumumab and panitumumab are approved for treatment of non-small-cell lung carcinoma and metastatic colorectal cancer [22, 23]. Cetuximab is approved for head and neck cancer and colorectal cancer [19, 23]. Necitumumab is associated with infusion reactions, hypomagnesemia, diarrhea, and dermatological toxicities [22]. Panitumumab has grade 3 and grade 4 adverse events and a high incidence (90–100%) of acneiform rash [23] similar to cetuximab. In contrast, nimotuzumab has a much lower incidence (3%) of this type of rash and an absence of diarrhea or anaphylaxis, which one or both have been associated with EGFR targeting agents [20, 24].

The decreased toxicity of nimotuzumab is proposed to be due to the relatively high K_D_ value of nimotuzumab, which requires bivalent binding to accumulate on EGFR expressing cells. Normal tissues such as skin with low levels of EGFR expression are bound monovalently by nimotuzumab and thus are retained transiently. Tumors overexpressing EGFR are bound bivalently by nimotuzumab and this avidity causes it to accumulate in EGFR overexpressing tumors [18]. We observed a correlation between IRDye800CW-nimotuzumab affinity and EGFR expression for low and intermediate EGFR expressing cell lines, however, at higher EGFR expression levels a threshold was reached where no further increase in IRDye800CW-nimotuzumab binding was observed. We did not see a similar correlation between receptor density and *in vivo* IRDye800CW-nimotuzumab binding. DLD-1, which has intermediate EGFR expression had similar IRDye800CW-nimotuzumab uptake compared to A-431, which had 15-fold higher EGFR expression. Similar results were reported with IRDye800CW-cetuximab accumulation in intermediate and high EGFR expressing cell lines [25]. These results could be due to physiologic conditions unrelated to EGFR expression, such as differences in vasculature, perfusion, and necrosis, which will ultimately affect delivery of the antibody [26, 27].

The dose of IRDye800CW-nimotuzumab was chosen for imaging and no therapeutic or toxic effects were specifically examined or expected. IRDye800CW has a drug master file with the FDA and is safe (NOAEL) at doses of 20 mg/kg [28], which is > 500-fold higher than the conjugated dye concentration used in this study. A therapeutic dose of nimotuzumab (1200 mg per patient) has no significant toxicities [29]. In this study, we used a human equivalent dose [30] > 80-fold lower than the human therapeutic dose and no adverse effects on the mice were observed.

In summary, the preclinical imaging properties of IRDye800CW-nimotuzumab combined with its low clinical toxicity, make it a promising antibody for repurposing as a NIR fluorescent imaging probe.

## MATERIALS AND METHODS

### Cell lines

All cell lines were grown at 37°C with 5% CO_2_. All cell lines were obtained from ATCC. A-431, DLD-1, and MDA-MB-435 cells were maintained in 90% Roswell Park Memorial Institute medium (RPMI) + 10% FBS. T-47D were maintained in 90% RPMI + 10% FBS + 0.2 units/mL bovine insulin. HT-29 cells were maintained in 90% McCoy’s media. MDA-MB-468 and MCF7 cells were maintained in 90% Dulbecco’s modified eagles medium (DMEM).

### Reagents

Nimotuzumab was supplied by the Center of Molecular Immunology (CIM) (Havana, Cuba). IRDye800CW was provided by Li-Cor Biosciences (Lincoln, NE). Cetuximab was obtained from Royal University Hospital (RUH) Saskatoon Pharmacy. Anti-maltose binding protein (anti-MBP) Fab was a generous gift from Dr. Sidhu (University of Toronto, Canada) and was sub-cloned into pFUSE-ss-CHIg-hG1and pFUSE-CLIG-hk plasmids (InvivoGen) to produce the IgG1kappa [31]. Anti-MBP IgG was expressed using Expi293 expression system (Thermo Fisher Scientific, Waltham, MA) according to the manufacturer’s instructions. The IgG was purified on MabSelect SuRe column (GE Healthcare Life Sciences, Chicago, Il) according to the manufacturers’ instructions and dialysed overnight in phosphate buffered saline (PBS).

### Antibody labeling with IRDye® 800CW and characterization

One milligram of antibody was labeled in 1X PBS (pH 7.0) with IRDye^®^ 800CW NHS Ester (Li-Cor Biosciences, Lincoln, NE) at a 1:3 antibody:dye molar ratio at 4°C overnight according to the manufacturer’s instructions. After labeling the unconjugated dye was removed using a Zeba 7000 Da molecular weight cut-off desalting column (ThermoFisher Scientific, Waltham, MA). Labeled antibodies were filter sterilized using Ultrafree MC centrifugal filter unit (Millipore, Billerica, MA). The concentration of IRDye800CW-nimotuzumab was measured using the following formula,

$$\mathrm{Protein}\;\mathrm{Conc}\left(\frac{\mathrm{mg}}{\mathrm{ml}}\right)=\frac{\mathrm{A280}-\left(0.03\times \mathrm{A780}\right)}{\mathrm{\mu Protein}}\times \mathrm{MWprotein}\times \mathrm{dilution}\;\mathrm{factor}$$

In which, 0.03 is a correction factor for the absorbance of IRDye 800CW at 280 nm (equal to 3.0% of its absorbance at 780 nm). ε_Protein_ is the molar extinction coefficients for the protein. MW_protein_ is the molecular weight of the protein. Dilution factor is the dilution of the labeled conjugate prior to measurement by spectrophotometer. The degree of NIR conjugation, that is, NIR/protein molar ratio, was calculated with the NIR/protein = (A_789_/ε_IR_)/(A_280_ – (0.03 x A_778_)/ε_Protein_) where the molar extinction coefficient of NIR (ɛIR) is 240,000 M^−1^ cm^−1^ and the molar extinction coefficients for the proteins (ɛ_protein_) are 217, 190 M^-1^ cm^-1^ for nimotuzumab, 217,440 M^-1^ cm^-1^ for cetuximab and 215,180 M^-1^ cm^-1^ for MBP IgG.

All labeled and unlabeled proteins were analyzed for purity using 2.5 µg of the protein loaded on an Agilent 2100 Bioanalyzer (Agilent Technologies, Santa Clara, CA) according to the manufacturer’s instructions.

Binding kinetics between the antibodies and recombinant proteins were measured by BLI with ForteBio Octet RED384 (PALL Corporation, Port Washington, NY). Antibodies were immobilized on Anti-human FAB-CH1 sensors (Forte Bio, Menlo Park, CA) according to the manufacture’s instructions. After immobilization, antibodies were exposed to 500 nM, 166 nM, and 55 nM concentrations of the target proteins, human EGFR (Sino Biological, Beijing, China). At the same time, empty sensors were exposed to the same concentrations of the target protein to be used for subtraction of non-specific binding. All reactions were performed at 30°C in 1X kinetics buffer (Forte Bio, Menlo Park, CA). The equilibrium dissociation constant (K_D_) was obtained using a 1 to 1 model with global fitting. Data analysis and curve fitting was performed using Data Analysis software 7.1.0.33 (Forte Bio, Menlo Park, CA). A baseline was determined in 1X kinetics buffer for 5 minutes. The sensors were loaded for 5 minutes in 75 nM of antibody or 1X kinetics buffer (for negative subtraction). The second baseline was collected for 5 minutes in 1X kinetics buffer. Association was performed for 3 minutes in 500 nM, 166.7 nM, 55.5 nM of the target protein. Dissociation was determined in 1X kinetics buffer for 10 minutes after which the sensors were regenerated.

### *In vitro* binding by flow cytometry

*In vitro* binding studies of IRDye800CW-nimotuzumab and the control were done in EGFR-positive human cancer cells lines with A-431 (head and neck cancer), DLD-1 (colorectal cancer), and HT-29 (colorectal cancer), MDA-MB-468 (breast cancer), MCF7 and T-47D (breast cancer), MDA-MB-435 (melanoma). 1 x 10^5^ cells were collected and washed with 1X PBS + 2% FBS. Antibodies were titrated at a minimum of a 10-fold molar excess onto the cells at concentrations between 0–2 µM in a 12-point curve. The reactions were incubated for 30 minutes at room temperature followed by 15 minutes on ice. Cells were washed and suspended in a 1:50 dilution of FITC labeled Goat F(ab’)2 fragment anti-human IgG (H + L) antibody (Beckman Coulter, Brea, CA) and incubated for 30 minutes on ice in the dark. Cells were washed and suspended in 1X PBS + 2% FBS and analyzed using a Gallios flow cytometer (Beckman Coulter, Brea, CA) on the FL1 channel. FlowJo V.10.0.8 was used for analysis. To rank EGFR expression the change in mean fluorescence intensity (ΔMFI) was calculated as the ΔMFI (fitted max MFI – fitted min MFI) and indicates how much nimotuzumab can bind each cell-line. For flow cytometry analysis, the mean fluorescence intensity was determined using Flowjo software V.10.0.8. For fitting and normalization of the mean fluorescence intensity the top was globally fit and the bottom was fit to the average and then normalized. The K_D_ was calculated based on a non-linear regression curve using Prism6 version 6.

### Serum stability assay

IRDye800CW-Nimotuzumab IgG was incubated in mouse serum at a final concentration of 0.2 mg IgG per mL of serum at 37°C protected from light. Samples collected at 0, 24, 48, 72, and 168 h were run on sodium dodecyl sulphate polyacrylamide gel electrophoresis (SDS-PAGE) and imaged in the 800 channel on the Odyessy CLx NIR scanner (LI-COR, NE) to visualize the IRDye800CW-Nimotuzumab IgG band. Subsequently the gel was Coomassie Blue stained and rescanned with the 800 and 700 channel to visualize the serum proteins in the 700 fluorescence channel to evaluate loading and visualize the protein ladder. The relative fluorescence intensity of each time point IRDye800CW-nimotuzumab IgG band was normalized to the 0 h band intensity. Each sample was done in duplicate and analysis is the average of a minimum of four data points ±SD.

### Mouse xenograft models

CD-1 nude female mice (Charles River Laboratories, Hartford, CT), aged 4–6 weeks, were obtained and housed in accordance with the University Animal Care Committee (UACC) guidelines (protocol # 20150048). All experiments and euthanasia were performed in accordance with UACC guidelines. For xenograft preparation, 10 **×** 10^6^ cells A-431, DLD-1 or MDA-MB-435 were collected and washed with growth media without FBS. Cells were suspended in 50 µL of growth media without FBS plus 50 µL of matrigel membrane matrix (Corning, Corning NY) and cells suspended in 100 µL was injected subcutaneously into the right hind flank of the mouse. Tumor size was monitored weekly until they reached 150–300 mm^3^. The mice were then intravenously injected through the tail vein with 0.5 nmoles (75 µg) of IRDye-800CW-nimotuzumab, IRDye-800CW-cetuximab, IRDye800CW-control IgG or 1 nmole of quenched IRDye800CW free dye.

### Fluorescence imaging and *ex vivo* Biodistribution

Tumor bearing mice (*n* ≥ 3 per group) were injected via tail vein with 0.5 nmoles of IRDye800CW-nimotuzumab or the control IgG (75 µg IgG) antibodies formulated in PBS and imaged using a Pearl Impulse small animal imaging system (LI-COR, NE) at 1, 6, 24, 48, 72, 96, 168, and 504 hpi. Images of mice were analyzed using Image Studio Software (version 3.1). Regions of interest (ROI) for xenografts, liver, kidneys, contralateral side, and background were selected from equivalent-sized areas containing the same number of pixels. Quantification of uptake was done by drawing ROIs (three per organ) for each organ and mean uptake in the organs was determined using the following equation:

$$\text{Normalized fluorescence}\left(\text{au}\right)={\text{Signal}}_{\text{xenograft}}-{\text{Signal}}_{\text{contralateral}}/\text{Labeling ratio}$$

Where Signal_xenograft_ is the mean fluorescence from the three ROIs from the xenograft, the Signal_contralateral_ is the mean fluorescence from the three ROIs from the contralateral side and the labeling ratio is the labeling ratio of the protein. For biodistribution studies, at least three mice per group were injected via tail vein with 0.5 nmole each of IRDye800CW-nimotuzumab or the control IgG. Animals were imaged at different time intervals after injection and immediately before dissection and organ collection. Immediately after imaging, mice were weighed (Supplementary Figure 6) euthanized and all organs were collected and stored on ice. Dissected organs were immediately weighed in tared tubes and imaged using the Pearl Impulse small animal imaging system to obtain the mean (mean of two ROIs) MFI for the organ. Fluorescence in organs was expressed as mean fluorescence or signal/area using arbitrary units (AU). After imaging organs were weighed (Supplementary Figure 6) and stored at–80°C for homogenization and subsequent analysis.

Homogenization was performed as described by Oliveira *et al.* [32] with some modifications. Briefly, 40-60 mg of each organ was added to lysis matrix A (MP Biomedicals Santa Ana, CA) with 1 mL of RIPA buffer (50 mM Tris-HCl, 150 mM NaCl, 1% Igepal (NP40), 0.5% Na-deoxycholate, 0.1% SDS and 1% protease inhibitor cocktail (Roche). Samples were homogenized using a FastPrep120 pulsed for 30 seconds on, 5 minutes off three times and centrifuged for 5 minutes at 12,000 **×** g. 200 µL of homogenate was aliquoted into a black 96-well optical bottom plate (Fisher) and serial diluted in half with RIPA buffer 6 times. The plate was centrifuged for 10 minutes at 1000 **×** g and scanned on a Li-Cor Odyssey scanner and expressed as mean fluorescence (signal) per gram of tissue.

### Statistical analysis

To compare the K_D_ values from flow cytometry and Octet and ΔMFI from flow cytometry and nimotuzumab fluorescence in different cell lines we used one-way analysis of variance (ANOVA) with multiple comparisons using Prism6 version 6. The calculated K_D_s for labeled and unlabeled nimotuzumab and nimotuzumab compared to the control IgG in fluorescence imaging were compared with a parametric unpaired *t*-test using Prism6 version 6. All error bars are standard error of the mean (sem) unless otherwise noted.

## SUPPLEMENTARY MATERIALS FIGURES



## Abbreviations

(EGFR): Epidermal growth factor receptor
(NIR): Near infrared
(CT): Computed tomography
(PET): Positron emission tomography
(MRI): Magnetic resonance imaging
(MB): Methylene blue
(5-ALA): 5-aminolevulinic acid
(ICG): Indocyanine green
(FITC): Fluorescein
(GMP): Good manufacturing practice
(FRI): Fluorescence reflectance imaging
(FMT): Fluorescence molecular tomography
(BLI): Biolayer interferometry
(MBP): Maltose binding protein
(hpi): Hours post injection
(AU): Arbitrary units
(ROI): Regions of interest

## References

[1] Robinson DR, Wu YM, Lin SF (2000). The protein tyrosine kinase family of the human genome. Oncogene.

[2] Yewale C, Baradia D, Vhora I, Patil S, Misra A (2013). Epidermal growth factor receptor targeting in cancer: A review of trends and strategies. Biomaterials.

[3] Chung CH, Ely K, McGavran L, Varella-Garcia M, Parker J, Parker N, Jarrett C, Carter J, Murphy BA, Netterville J, Burkey BB, Sinard R, Cmelak A (2006). Increased epidermal growth factor receptor gene copy number is associated with poor prognosis in head and neck squamous cell carcinomas. J Clin Oncol.

[4] Alshenawy HA (2010). Immunohistochemical expression of epidermal growth factor receptor, e-cadherin, and matrix metalloproteinase-9 in ovarian epithelial cancer and relation to patient deaths. Ann Diagn Pathol.

[5] Cunningham D, Humblet Y, Siena S, Khayat D, Bleiberg H, Santoro A, Bets D, Mueser M, Harstrick A, Verslype C, Chau I, Van Cutsem E (2004). Cetuximab monotherapy and cetuximab plus irinotecan in irinotecan-refractory metastatic colorectal cancer. N Engl J Med.

[6] Giltnane JM, Rydén L, Cregger M, Bendahl PO, Jirström K, Rimm DL (2007). Quantitative measurement of epidermal growth factor receptor is a negative predictive factor for tamoxifen response in hormone receptor positive premenopausal breast cancer. J Clin Oncol.

[7] Bellone S, Frera G, Landolfi G, Romani C, Bandiera E, Tognon G, Roman JJ, Burnett AF, Pecorelli S, Santin AD (2007). Overexpression of epidermal growth factor type-1 receptor (EGF-R1) in cervical cancer: Implications for cetuximab-mediated therapy in recurrent/metastatic disease. Gynecol Oncol.

[8] Pleijhuis RG, Graafland M, de Vries J, Bart J, de Jong JS, van Dam GM (2009). Obtaining adequate surgical margins in breast-conserving therapy for patients with early-stage breast cancer: Current modalities and future directions. Ann Surg Oncol.

[9] van Dam GM, Themelis G, Crane LM, Harlaar NJ, Pleijhuis RG, Kelder W, Sarantopoulos A, de Jong JS, Arts HJ, van der Zee AG, Bart J, Low PS, Ntziachristos V (2011). Intraoperative tumor-specific fluorescence imaging in ovarian cancer by folate receptor-α targeting: First in-human results. Nat Med.

[10] Saccomano M, Dullin C, Alves F, Napp J (2016). Preclinical evaluation of near-infrared (NIR) fluorescently labeled cetuximab as a potential tool for fluorescence-guided surgery. Int J Cancer.

[11] Stummer W, Pichlmeier U, Meinel T, Wiestler OD, Zanella F, Reulen HJ, ALA-Glioma Study Group (2006). Fluorescence-guided surgery with 5-aminolevulinic acid for resection of malignant glioma: A randomised controlled multicentre phase III trial. Lancet Oncol.

[12] Warram JM, de Boer E, Sorace AG, Chung TK, Kim H, Pleijhuis RG, van Dam GM, Rosenthal EL (2014). Antibody-based imaging strategies for cancer. Cancer Metastasis Rev.

[13] Kosaka N, Ogawa M, Choyke PL, Kobayashi H (2009). Clinical implications of near-infrared fluorescence imaging in cancer. Future Oncol.

[14] Day KE, Sweeny L, Kulbersh B, Zinn KR, Rosenthal EL (2013). Preclinical comparison of near-infrared-labeled cetuximab and panitumumab for optical imaging of head and neck squamous cell carcinoma. Mol Imaging Biol.

[15] Heath CH, Deep NL, Sweeny L, Zinn KR, Rosenthal EL (2012). Use of panitumumab-irdye800 to image microscopic head and neck cancer in an orthotopic surgical model. Ann Surg Oncol.

[16] Korb ML, Hartman YE, Kovar J, Zinn KR, Bland KI, Rosenthal EL (2014). Use of monoclonal antibody-irdye800cw bioconjugates in the resection of breast cancer. J Surg Res.

[17] Available from: clinicaltrials.gov. Accessed 24 May 2017

[18] Garrido G, Tikhomirov IA, Rabasa A, Yang E, Gracia E, Iznaga N, Fernández LE, Crombet T, Kerbel RS, Pérez R (2011). Bivalent binding by intermediate affinity of nimotuzumab: A contribution to explain antibody clinical profile. Cancer Biol Ther.

[19] Bonner JA, Harari PM, Giralt J, Azarnia N, Shin DM, Cohen RB, Jones CU, Sur R, Raben D, Jassem J, Ove R, Kies MS, Baselga J (2006). Radiotherapy plus cetuximab for squamous-cell carcinoma of the head and neck. N Engl J Med.

[20] Ramakrishnan MS, Eswaraiah A, Crombet T, Piedra P, Saurez G, Iyer H, Arvind AS (2009). Nimotuzumab, a promising therapeutic monoclonal for treatment of tumors of epithelial origin. mAbs.

[21] Thomas SM, Grandis JR (2004). Pharmacokinetic and pharmacodynamic properties of EGFR inhibitors under clinical investigation. Cancer Treat Rev.

[22] Brinkmeyer JK, Moore DC (2018). Necitumumab for the treatment of squamous cell non-small cell lung cancer. J Oncol Pharm Pract.

[23] Price TJ, Peeters M, Kim TW, Li J, Cascinu S, Ruff P, Suresh AS, Thomas A, Tjulandin S, Zhang K, Murugappan S, Sidhu R (2014). Panitumumab versus cetuximab in patients with chemotherapy-refractory wild-type KRAS exon 2 metastatic colorectal cancer (ASPECCT): A randomised, multicentre, open-label, non-inferiority phase 3 study. Lancet Oncol.

[24] Allan DG (2005). Nimotuzumab: Evidence of clinical benefit without rash. Oncologist.

[25] Aerts HJ, Dubois L, Perk L, Vermaelen P, van Dongen GA, Wouters BG, Lambin P (2009). Disparity between *in vivo* EGFR expression and 89zr-labeled cetuximab uptake assessed with PET. J Nucl Med.

[26] Viloria-Petit A, Crombet T, Jothy S, Hicklin D, Bohlen P, Schlaeppi JM, Rak J, Kerbel RS (2001). Acquired resistance to the antitumor effect of epidermal growth factor receptor-blocking antibodies *in vivo*: A role for altered tumor angiogenesis. Cancer Res.

[27] Lu Y, Li X, Liang K, Luwor R, Siddik ZH, Mills GB, Mendelsohn J, Fan Z (2007). Epidermal growth factor receptor (EGFR) ubiquitination as a mechanism of acquired resistance escaping treatment by the anti-egfr monoclonal antibody cetuximab. Cancer Res.

[28] Marshall MV, Draney D, Sevick-Muraca EM, Olive DM (2010). Single-dose intravenous toxicity study of IRDye 800CW in Sprague-Dawley rats. Mol Imaging Biol.

[29] Wang C, Fu X, Cai X, Wu X, Hu X, Fan M, Xiang J, Zhang Y, Chen H, Jiang G, Zhao K (2015). High-dose nimotuzumab improves the survival rate of esophageal cancer patients who underwent radiotherapy. Onco Targets Ther.

[30] Nair AB, Jacob S (2016). A simple practice guide for dose conversion between animals and human. J Basic Clin Pharm.

[31] Karauzum H, Chen G, Abaandou L, Mahmoudieh M, Boroun AR, Shulenin S, Devi VS, Stavale E, Warfield KL, Zeitlin L, Roy CJ, Sidhu SS, Aman MJ (2012). Synthetic human monoclonal antibodies toward staphylococcal enterotoxin B (SEB) protective against toxic shock syndrome. J Biol Chem.

[32] Oliveira S, Cohen R, Walsum MS, van Dongen GA, Elias SG, van Diest PJ, Mali W, van Bergen En Henegouwen PM (2012). A novel method to quantify irdye800cw fluorescent antibody probes *ex vivo* in tissue distribution studies. EJNMMI Res.

