# Supplementary material for: Near infrared fluorescence imaging of EGFR expression in vivo using IRDye800CW-nimotuzumab
Source: Oncotarget. 2017 Dec 21;9(5):6213–27. doi: 10.18632/oncotarget.23557 (PMC5814206; doi:10.18632/oncotarget.23557)
Supplement: Supplementary file 1 [file oncotarget-09-6213-s001.pdf]

Near infrared fluorescence imaging of EGFR expression *in vivo* using IRDye800CW-nimotuzumab

SUPPLEMENTARY MATERIALS

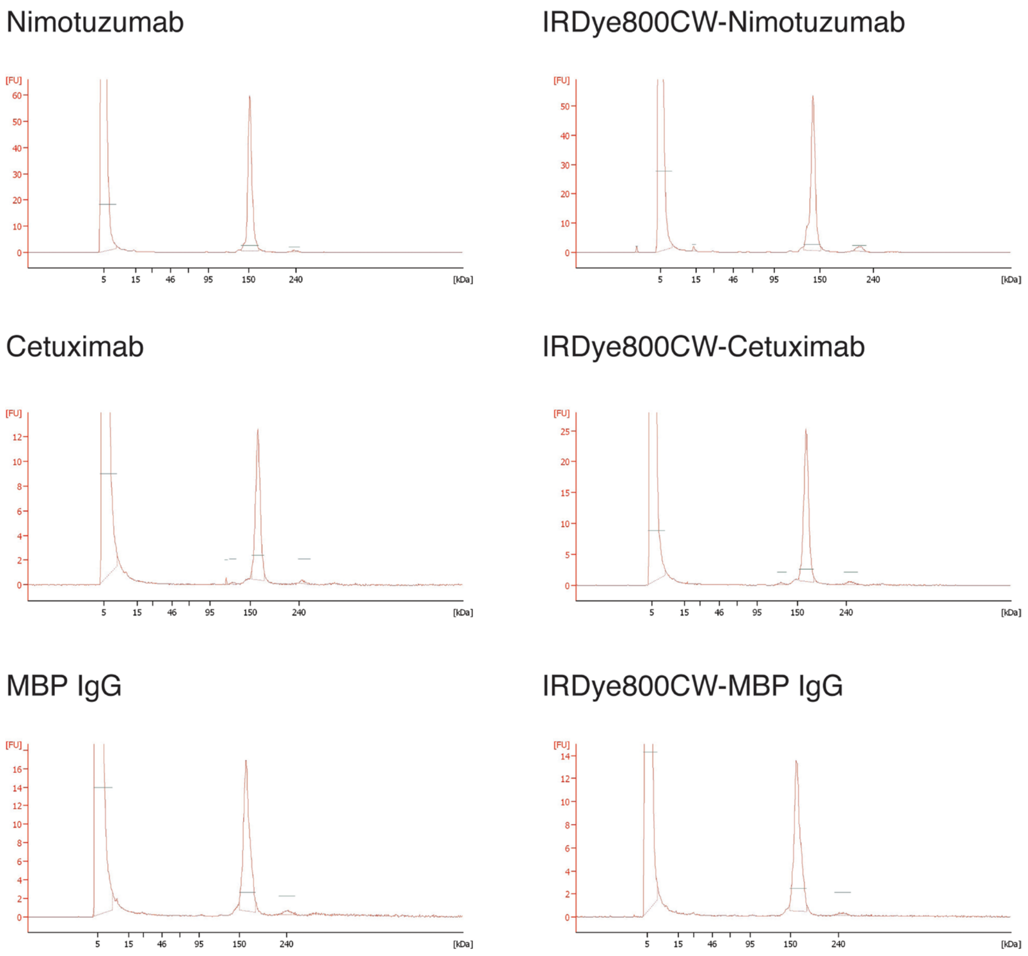

Construct	Size (kDa)	Purity (%)
Nimotuzumab	144.2	99
IRDye800CW-nimotuzumab	149.5	95
Cetuximab	164.1	96
IRDye800CW-cetuzimab	165.7	97
MBP IgG	160	97
IRDye800CW-MBP IgG	161.8	98

Supplementary Figure 1: Characterization of nimotuzumab, cetuximab, and the control IgG using the Agilent Bioanalyzer 2100. Unlabeled and IRDye800CW- labeled nimotuzumab, cetuximab, and the control IgG were analysed by Agilent Bioanalyzer 2100. The size in kDa and purity (%) are shown.

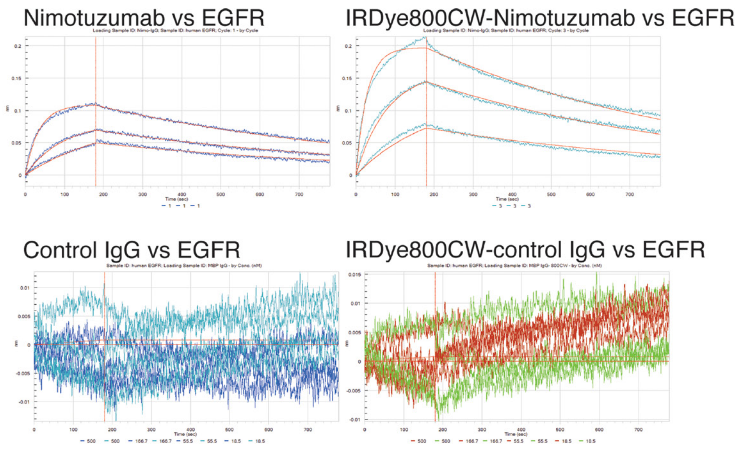

Target (EGFR)	Antibody	KD (nM)	SD (nM)	Association (1/Ms)	Association SD (1/Ms)	Dissociation (1/s)	Dissociaiton SD (1/s)
Human	Nimotuzumab	22.3	2.3	6.1×10^4	8.6×10^3	1.3×10^3	6.4×10^5
Human	Nimotuzumab-IRDye800CW	20.1	0.8	6.5×10^4	6.7×10^3	1.3×10^3	1.9×10^4
Human	MBP IgG	NB *	-	-	-	-	-
Human	MBP IgG-IRDye800CW	NB	-	-	-	-	-

* NB = no binding, SD = standard deviation

Supplementary Figure 2: Biolayer interferometry analysis of binding kinetics of nimotuzumab and the control IgG. Unlabeled and IRDye800CW-labeled nimotuzumab and the control IgG binding kinetics against human EGFR.

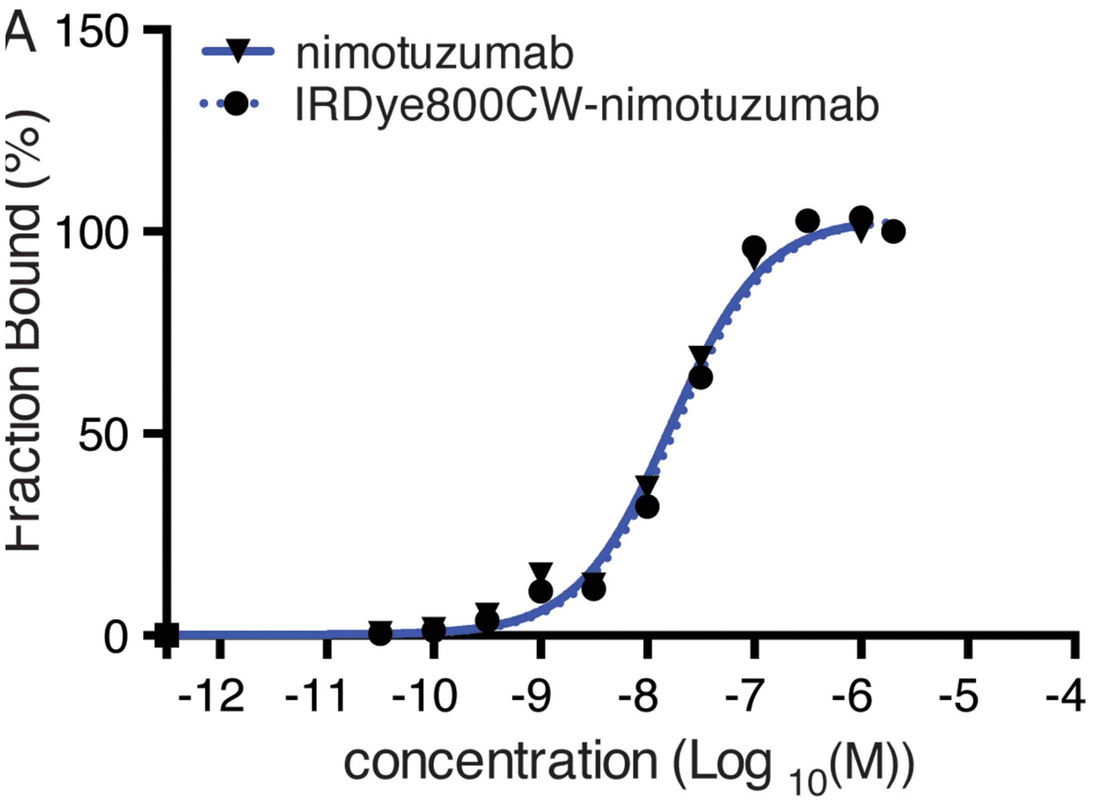

Supplementary Figure 3: Flow cytometry binding of nimotuzumab and IRDye800CW-nimotuzumab to A431 cells.

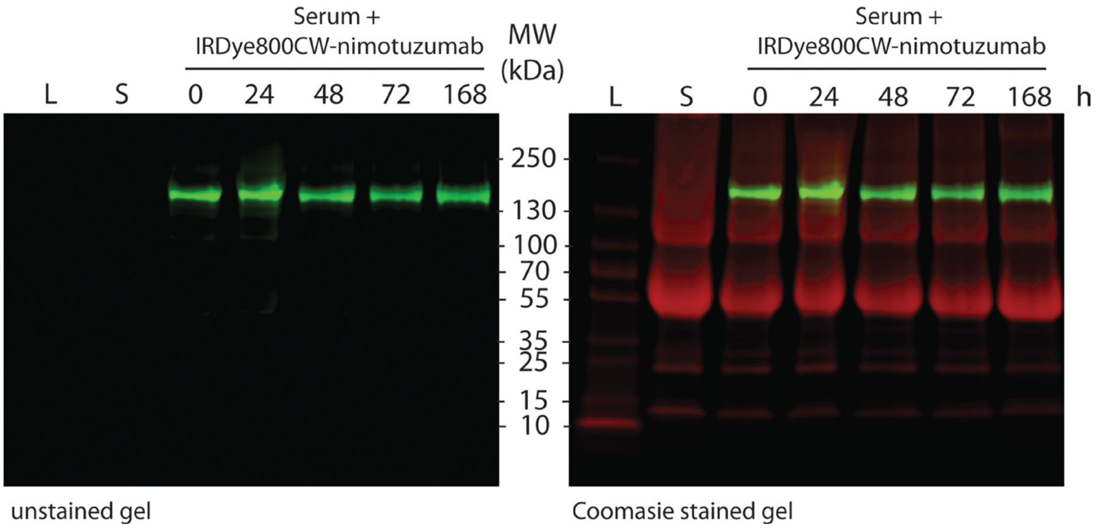

Supplementary Figure 4: Serum stability of IRDye800CW-nimotuzumab. Representative SDS-PAGE gel showing IRDye800CW-nimotuzumab in the 800 channel (green) at various time points (0, 24, 48, 72 and 168 hours). Coomassie stained gel stains serum proteins and the ladder and is visible in the 700 channel (red). L = Ladder, S = Serum alone.

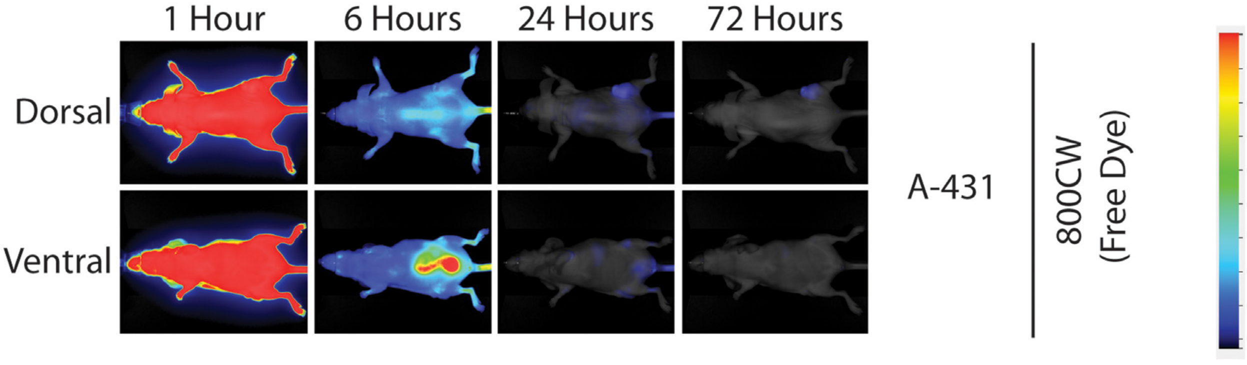

Supplementary Figure 5: Near infrared imaging of free IRDye800CW. Mice were intravenously injected with 1 nmole of IRDye800CW and fluorescent images were taken in mice bearing A-431 xenografts. Images shown were taken at 1, 6, 24, and 72 hours post injection. Fluorescent scale is shown on the right. Xenograft is in right flank (top) of mouse in dorsal images.

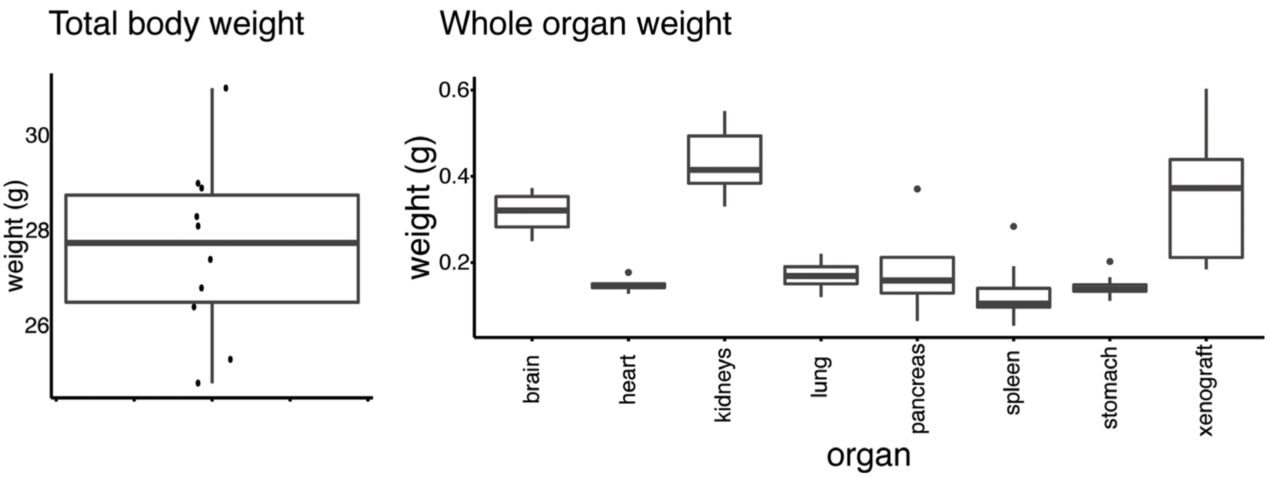

Supplementary Figure 6: Mouse total body weights and the whole organ weights used for biodistribution analysis.
